# Supplementary material for: Characterization of the Immune Response to PD-1 Blockade during Chemoradiotherapy for Head and Neck Squamous Cell Carcinoma
Source: Cancers (Basel). 2022 May 19;14(10):2499. doi: 10.3390/cancers14102499 (PMC9139476; doi:10.3390/cancers14102499)
Supplement: Supplementary file 1 [file cancers-14-02499-s001.zip › cancers-1697111-supplementary.pdf]

Table S1. Antibodies used for 3 panel stain to identify immune cell subsets in murine tumor samples. Panel 1: CD4+ T cells, CD8+ T cells, Treg and Immune checkpoint proteins; Panel 2: MDSC-like. Intracellular antibodies are underlined, and clones and company are listed under each antibody.

Fluorochrome	Panel 1	Panel 2
**Blue Dead Cell Stain**	Live/dead Invitrogen	Live/dead Invitrogen
**BV510**	CD3 (17A2 Biolegend)	CD3 (17A2 Biolegend)
**BV605**	CD4 (RM4-5 Biolegend)	-
**APC-Cy7**	CD8 (53-6.7 Biolegend)	-
**PE**	FOXP3 (3G3 Thermo)	-
**APC**	Ki67 (SolA15 Thermo)	MHCII (M5 eBioscience)
**AF488**	CD45 (104 Biolegend)	CD45 (104 Biolegend)
**BV711**	PD-1 (29F.1A12 Biolegend)	-
**BV421**	LAG-3 (C9B7W Biolegend)	-
**PerCP/Cy5.5**	TIM-3 (B8.2C12 Biolegend)	Ly-6C (HK1.4 Biolegend)
**PE**	-	CD11b (M1/70 eBioscience)
**AF700**	-	Ly-6G (1A8 Biolegend)

FoxP3, forkhead box P3; MHCII, major histocompatibility complex II; MDSC, myeloid derived suppressor cells; PD1, programmed cell death 1; TIM3, T cell immunoglobulin and mucin domain 3; LAG3, Lymphocyte-activation gene 3; Treg, regulatory T cells.

Table S2. Antibodies used for 3 panel stain to identify peripheral immune cell subsets. Panel 1: Immune checkpoint proteins; Panel 2: CD4+ T cells, CD8+ T cells and Treg; Panel 3: B cells and MDSC-like. Intracellular antibodies are underlined, and clones and company are listed under each antibody.

Fluorochrome	Panel 1	Panel 2	Panel 3
**Blue Dead Cell Stain**	Live/dead Invitrogen	Live/dead Invitrogen	Live/dead Invitrogen
**PerCP/CY5.5**	CD3 (UCHT1 Biolegend)	CD3 (UCHT1 Biolegend)	CD3 (UCHT1 Biolegend)
**BV605**	CD4 (RPA-T4 BD)	CD4 (RPA-T4 BD)	-
**APC-Cy7**	CD8 (SK1 BD)	CD8 (SK1 BD)	-
**PE-CY7**	CCR7 (G043H7 Biolegend)	-	HLA DR (C243 Biolegend)
**BV421**	CD45RA (HI100 Biolegend)	CD45RA (HI100 Biolegend)	CD33 (WM53 BD)
**FITC**	PD-1 (EH12.2 H7 Biolegend)	CD127 (eBioRDR5	CD15 (HI98 Biolegend)
**PE**	TIM-3 (CD366 BD)	CD25 (M-A251 Biolegend)	-
**A647**	LAG-3 (CD223 BD)	FOXP3 (206D Biolegend)	-
**AF700**	-	-	CD11b (ICRF44 BD)
**BV510**	-	-	CD14 (M5E2 Biolegend)
**BV737**	-	-	CD19 (SJ25C1 BD)

FoxP3, forkhead box P3; HLA, human leukocyte antigen; M-MDSC, monocytic myeloid derived suppressor cells; PD-1, programmed cell death 1; TIM3, T cell immunoglobulin and mucin domain 3; LAG3, Lymphocyte-activation gene 3; Treg, regulatory T cells.

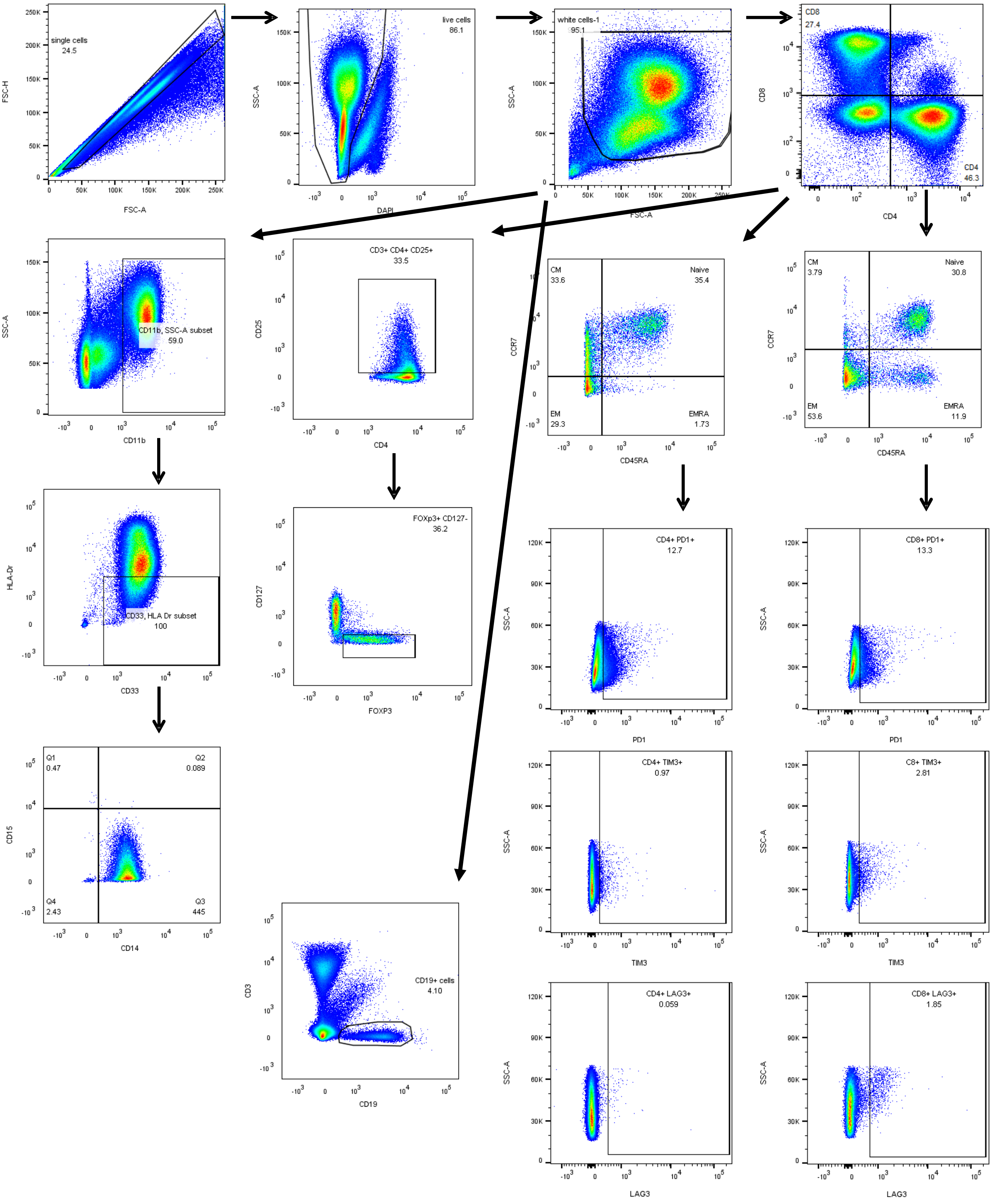

Figure S1. Gating strategy to identity immune cell subsets. Three immune flow cytometry panels using PBMC from a cancer patients were used. Classic immune cell types included CD4+ T cells, CD8+ T cells, Treg, B cells, and myeloid derived suppressor-like cells (MDSC-like).

Supplementary Methods S1: Sample size and statistical analysis for animal experiments.

Sample size estimates were calculated using a one-sided, two-arm binomial test with a power of 0.80 and type I error of 0.05. For monotherapy (control versus anti-PD-1) studies, sample size analysis was based on prior data demonstrating death (due to disease or meeting sacrifice criteria) of nearly all animals by 30 days with no treatment. To minimize animal use, due to low anticipated survival rates (null hypothesis <5% surviving at day 30) with control treatment, we hypothesized that if 50% or more animals were alive at day 30, then the anti-PD-1 treatment would be considered efficacious. As a result, a sample size of 10 for each group was needed to reject the null hypothesis. For combination therapy (chemoradiation plus IgG1 isotype control versus chemoradiation plus anti-PD-1), prior data indicate that survival at 100 days with control treatment (chemoradiation) is approximately 10% (null hypothesis) when treatment is initiated at 12 days post tumor implantation. With the addition of anti-PD-1 therapy to this treatment, we hypothesized that an improvement to 50% or more surviving is worthy of future study. This allowed for a sample size n = 15 in each treatment group to reject the null hypothesis.
